# Association between monocyte-lymphocyte ratio and all-cause and cardiovascular mortality in patients with chronic kidney diseases: A data analysis from national health and nutrition examination survey (NHANES) 2003-2010

**DOI:** 10.1080/0886022X.2024.2352126

**Published:** 2024-06-04

**Authors:** Wenwu Liu, Shuwei Weng, Chenghui Cao, Yuting Yi, Yue Wu, Daoquan Peng

**Affiliations:** Department of Cardiovascular Medicine, Research Institute of Blood Lipids and Atherosclerosis, The Second Xiangya Hospital, Central South University, Changsha, Hunan, China

**Keywords:** Chronic kidney disease, monocyte to lymphocyte ratio, all-cause mortality, cardiovascular mortality, NHANES

## Abstract

**Background:**

The relationship between monocyte-to-lymphocyte ratio (MLR) and prognosis in patients with chronic kidney disease (CKD) remains unclear. The aim of this study was to investigate the association between MLR and both all-cause mortality and cardiovascular disease (CVD) mortality in patients with CKD.

**Methods:**

This study analyzed data from National Health and Nutrition Examination Survey 2003-2010. This study included 11262 eligible subjects, and 3015 of them were with CKD. We first compared the differences in clinical characteristics between individuals with and without CKD, and then grouped the CKD population based on quartiles of MLR. The partial correlation analysis was conducted to assess the relationships between MLR and some important clinical features. Cox proportional hazards models were used to investigate the associations between MLR and mortality from all-cause and cardiovascular disease. Restricted cubic spline (RCS) was used to investigate the dose-response relationship between MLR and mortality, the receiver operating characteristic (ROC) curves is used to compare the efficacy of MLR with different clinical biological indicators in assessing the risk of death.

**Results:**

During a median follow-up of 10.3 years in CKD population, 1398 (43%) all-cause deaths and 526 (16%) CVD deaths occurred. It has been found that individuals with CKD have higher MLR level. The partial correlation analysis results showed that even after adjusting for age, sex, and race, MLR is still correlated with blood glucose, lipid levels, and kidney function indicators. The results of the cox proportional hazards regression model and Kaplan-Meier curve shown after adjusting for covariates, higher MLR was significantly associated with an increased risk of mortality. Consistent results were also observed when MLR was examined as categorical variable (quartiles). The RCS demonstrated a positive association between MLR and the risk of all-cause mortality and cardiovascular mortality. The ROC results indicate that the predictive efficacy of MLR for all-cause mortality risk is comparable to eGFR, higher than NLR and CRP. The predictive efficacy of MLR for cardiovascular mortality risk is higher than these three indicators.

**Conclusion:**

Compared to non-CKD population, the CKD population has higher levels of MLR. In the CKD population, MLR is positively correlated with the risk of death. Furthermore, the predictive efficacy of MLR for mortality risk is higher than other clinical indicators. This suggests that MLR can serve as a simple and effective clinical indicator for predicting mortality risk in CKD patients.

## Introduction

Today, 60% of all fatalities are caused by chronic diseases, making them an important problem over the world [1]. The prevalence of chronic kidney disease is estimated to be between 5% and 7% of the world’s population, with the prevalence being higher in developing nations [2]. Patients with renal failure typically have higher rates of morbidity and mortality due to cardiovascular disease. Patients with CKD are more likely to experience cardiac failure than people in general, and it is a reliable indicator of mortality in this population [3].

Chronic kidney disease typically progresses to a uremic state due to irreversible loss of nephrons, in which inflammation and activation of the immune system play crucial roles. Pro-inflammatory cells contribute to kidney function decline by releasing inflammatory factors. Some systemic inflammatory markers have been shown to be independent risk factors for CKD mortality, such as interleukin-6 and C-reactive protein (CRP) [4]. However, these indicators are difficult to routinely measure in primary healthcare settings, therefore selecting simpler inflammatory markers to predict CKD risk is of great importance.

MLR is a new inflammatory marker calculated from routine blood parameters, which can be used to reflect the overall level of inflammation in the body. Recent studies have found that MLR can be used to predict the new-onset of CKD [5]. Another small-sample cohort study demonstrated that a higher MLR is a stronger and independent predictor of mortality in hemodialysis patients [6]. However, there is a lack of evidence regarding the relationship between MLR and mortality risk in CKD patients. Therefore, this study aims to explore the association between MLR and the risk of all-cause mortality and cardiovascular mortality in CKD patients.

## Materials and methods

### Study population

The data we used were from National Health and Nutrition Examination Survey 2003-2010. NHANES participants had a standardized physical examination at a mobile examination center (ECM) by professionals. All participants gave written informed permission and more information is publicly available on the NHANES website.

**Figure 1.**
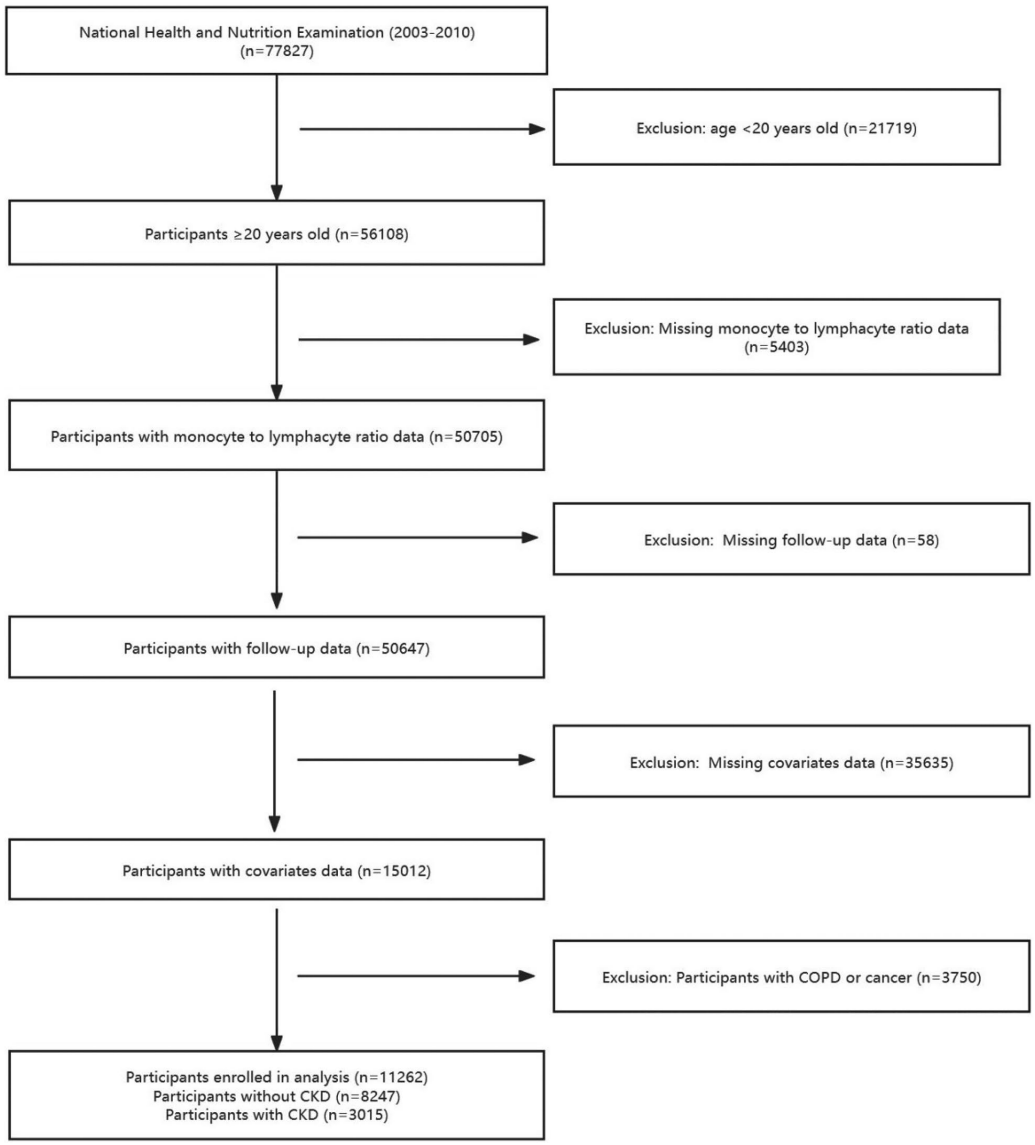
Flow diagram of the selection of eligible participants.

According to the analytical guideline, we merged the data from the NHANES 2003-2010. There are 77827 participants in the NHANES 2003-2010, we excluded the participants younger than 20 years (*n* = 21719), 56108 individuals were aged ≥20 years. We further excluded individuals with missing information on monocyte to lymphocyte ratio (*n* = 5403), subjects without follow-up data (*n* = 58) and other covariates (*n* = 35635). Considering the impact of diseases such as COPD and tumors on the body’s immune levels, we excluded patients with COPD and tumors (*n* = 3750). Finally, 11262 participants were included in our analysis, including individuals without CKD (*n* = 8247) and those with CKD (*n* = 3015) (defined as an estimated glomerular filtration rate (eGFR) <60 mL/min/1.73m^2^, using the CKD Epidemiology Collaboration equation, and/or a urinary albumin-Creatine ratio (ACR) >30mg/g) (Figure 1).

### Measurement of MLR and outcomes

Monocyte count to lymphocyte count ratio was used to determine MLR; both values may be acquired directly from laboratory data files. A blood sample was collected from the antecubital vein of all participants by a trained phlebotomist using MEC. Detailed information regarding laboratory testing can be found in the NHANES Laboratory/Medical Technician Procedures Manual. We track cardiovascular and all-cause mortality as the outcome. Up to December 31, 2019, participants were monitored unless they dropped out or achieved the goal. Participants’ mortality status was verified by comparison to data from the National Death Index. Cardiovascular mortality was defined as fatalities brought on by cerebrovascular disease or cardiovascular disease using the Clinical Modification System codes from the International Classification of Diseases, 10th Edition (ICD-10) (ICD-10 codes I00 to I09, I11, I13, I20 to I51, and I60 to I69).

### Covariates

The following covariates we selected were seen as confounding factors, including age, sex, race, education level, smoking status, body mass index (BMI), red blood cell (RBC), hemoglobin, alanine aminotransferase (ALT), serum creatine, blood urea nitrogen (BUN), total cholesterol (TC), triglyceride, non-HDL-C (non-HDL-C is calculated by subtracting high density lipoprotein cholesterol (HDL) from total cholesterol), glucose, glycated hemoglobin (HbA1c), albumin, CRP, neutrophil lymphocyte ratio (NLR), medication history of aspirin, statin, angiotensin-converting enzyme inhibitors (ACEI), status of diabetes, hypertension and CVD. Individuals whose averaged systolic blood pressure (SBP) of ≥140mmHg or a measured averaged diastolic blood pressure (DBP) of ≥90mmHg, or diagnosed hypertension and had antihypertensive drugs were seen as with hypertension. Participants whose fasting plasma glucose ≥7.0 mmol/L or glycosylated hemoglobin (HbA1c) ≥6.5% and those with diagnosed diabetes were defined as diabetes. CVD was defined as the existence of any type of following diseases, including congestive heart failure, coronary heart disease, angina pectoris, heart attack and stroke. If the participant answers "Yes" to the question "In the past 12 months, have you received dialysis (either hemodialysis or peritoneal dialysis)?", then they are considered to have received dialysis. Renal function is classified into 5 stages based on eGFR (G1: eGFR > 90 mL/min/1.73m^2^, G2: 60 < eGFR < 90 mL/min/1.73m^2^, G3: 30 < eGFR < 60 mL/min/1.73m^2^, G4: 15 < eGFR < 30 mL/min/1.73m^2^, G5: eGFR < 15 mL/min/1.73m^2^. Albuminuria is defined as an albumin-to creatinine ratio of 30 mg/g or higher, with microalbuminuria defined as an albumin-to-creatinine ratio of 30 mg/g to 299 mg/g, and macroalbuminuria defined as an albumin-to-creatinine ratio of 300 mg/g or higher

### Statistical analysis

In all of our analyses, we have considered sample weights to obtain national estimates. Additionally, a test for normality was conducted on the continuous variables. As the continuous variables did not conform to a normal distribution, they were represented using the median (interquartile range). Categorical variables were expressed as frequencies and percentages. A t-test was employed to compare the CKD population with the control group, while a one-way analysis of variance (ANOVA), Kruskal-Wallis H test, or chi-square test was used to assess differences among different quartile groups of MLR. Weighted cox regression models were constructed to explore the association between MLR and mortality risk instead of traditional cox regression, which was expressed as calculated hazard ratios (HRs) and 95% confidence interval (95% CIs). Crude analysis adjusted for no covariate, adjusted model adjusted for age, sex, race, education level, smoking status, hypertension, diabetes, CVD, history of statin and ACEI (model 1), and further adjust for BMI, SBP, ALT, TC, HbA1c, eGFR, BUN, albumin and CRP (model 2). Kaplan-Meier survival analyses were applied to analyze the differences of survival rates according to MLR groups, and the differences were examined by log-rank test. To investigate the dose-response relationship between MLR and mortality, a restricted cubic spline regression with the aforementioned multivariable adjustments was utilized. Two-tailed p-values of < 0.05 were considered statistically significant.

## Results

### Baseline characteristics of study population

A total of 11262 NHANES participants were included in this study. The baseline features of all the eligible participants are presented in Table 1. Among them, the medium age was 51 years old, the male accounted for 47%, and the weighted prevalence of hypertension was 21%. The median value of MLR was 0.27, and the individuals with CKD tended to have higher MLR relative to those without CKD. We also identified that CKD individuals have higher BMI, SBP, BUN, serum creatinine, HbA1c, glucose, triglyceride, and CRP levels, but lower hemoglobin, ALT, eGFR, TC, albumin levels. The overall prevalence rates of hypertension, diabetes, CVD, heart failure, CHD were 48%, 23%, 15%, 3.9%, and 6.4% respectively, and people with CKD seemed to have a higher risk of them. However, there is a trend of increased dialysis rate in CKD patients, but no significant difference between the two groups. CKD individuals had a higher mortality than those without CKD. Then, we divided the CKD individuals according to the MLR quartiles and compared the differences of the above indicators among the groups. The results are shown in Supplementary Table 1. Participants with higher MLR levels were more likely to be male and White; and those had higher creatine, BUN, NLR and lower eGFR, TC, TG levels (all *p* < 0.05), During a median follow-up of 10.3 years, the all-cause and CVD mortality were 43% and 16%, In CKD patients, the higher the level of MLR, the greater the risk of all-cause mortality and cardiovascular mortality.

**Table 1.** Clinical features of the participants with or without CKD.

Characteristic	Overall (*n* = 11262)	Non-CKD (*n* = 8247)	CKD (*n* = 3015)	P Value
Age (years)	51 (40, 64)	49 (37, 60)	66 (53, 77)	<0.001
Gender (male, %)	5,379 (47%)	3,856 (47%)	1,523 (46%)	0.7
Race				0.4
Mexican American	1,920 (7.0%)	1,424 (7.0%)	496 (6.9%)	
Non-Hispanic Black	2,097 (10%)	1,543 (10.0%)	554 (11%)	
Non-Hispanic White	5,716 (74%)	4,072 (73%)	1,644 (75%)	
Other Race	1,529 (9.4%)	1,208 (NA%)	321 (NA%)	
Education level				<0.001
College or above	5,111 (55%)	4,053 (59%)	1,058 (42%)	
High School	2,749 (26%)	1,968 (25%)	781 (31%)	
Less than high school	3,402 (19%)	2,226 (17%)	1,176 (27%)	
Smoking status (%)				0.4
Current smoker	2,195 (20%)	1,723 (21%)	472 (17%)	
Former smoker	3,226 (28%)	2,198 (28%)	1,028 (30%)	
Never smoker	5,841 (52%)	4,326 (52%)	1,515 (52%)	
Comorbidity				
Hypertension, n (%)	6,187 (48%)	3,789 (41%)	2,398 (77%)	<0.001
Diabetes, n (%)	3,366 (23%)	1,753 (17%)	1,613 (45%)	<0.001
CVD, n (%)	2,143 (15%)	1,067 (9.9%)	1,076 (32%)	<0.001
Heart failure, n (%)	617 (3.9%)	237 (2.0%)	380 (11%)	<0.001
CHD, n (%)	888 (6.4%)	466 (4.5%)	422 (14%)	<0.001
Dialysis	69 (8.4%)	8 (5.5%)	61 (9.5%)	0.6
Medication history				
Statin	750 (6.3%)	496 (5.8%)	254 (8.2%)	<0.001
Aspirin	157 (1.0%)	89 (0.8%)	68 (2.0%)	<0.001
ACEI	620 (4.9%)	415 (4.5%)	205 (6.5%)	<0.001
ACR (mg/g)				<0.001
< =30	9,255 (87%)	8,247 (100%)	1,008 (35%)	
30-300	1,841 (12%)	0 (0%)	1,841 (60%)	
> =300	166 (1.0%)	0 (0%)	166 (4.6%)	
eGFR (ml/min/1.73 m2)				<0.001
G1 (>90)	5,805 (53%)	5,048 (59%)	757 (28%)	
G2 (60-90)	3,804 (36%)	3,199 (41%)	605 (19%)	
G3a (45-59)	1,026 (7.3%)	0 (0%)	1,026 (35%)	
G3b (30-44)	372 (2.2%)	0 (0%)	372 (11%)	
G4 (15-30)	211 (1.4%)	0 (0%)	211 (6.5%)	
G5 (<15)	44 (0.2%)	0 (0%)	44 (0.8%)	
eGFR (ml/min/1.73 m2)	92 (75, 108)	95 (82, 109)	59 (51, 92)	<0.001
Body mass index (kg/m^2^)	28 (25, 33)	28 (25, 33)	30 (26, 35)	<0.001
Systolic blood pressure (mmHg)	120 (110, 132)	119 (109, 129)	130 (116, 144)	<0.001
Red blood cell (10^9^/L)	4.65 (4.32, 5.02)	4.69 (4.39, 5.03)	4.51 (4.07, 4.98)	<0.001
hemoglobin (g/dL)	14.30 (13.30, 15.30)	14.40 (13.40, 15.40)	13.90 (12.60, 15.00)	<0.001
Platelet (10^9^/L)	250 (209, 297)	252 (213, 298)	242 (201, 287)	0.001
ALT (U/L)	22 (17, 29)	22 (17, 29)	21 (17, 27)	0.005
Serum creatine (umol/L)	0.89 (0.74, 1.00)	0.83 (0.72, 0.97)	1.06 (0.86, 1.32)	<0.001
Blood urea nitrogen (mmol/L)	4.64 (3.57, 5.71)	4.28 (3.57, 5.36)	6.07 (4.28, 8.21)	<0.001
TC (mg/dL)	189 (165, 218)	192 (167, 221)	180 (153, 206)	<0.001
TG (mg/dL)	115 (81, 171)	112 (78, 165)	127 (92, 197)	<0.001
Non-HDL-C	135 (110, 164)	137 (113, 165)	126 (100, 157)	<0.001
Glucoses (mmol/l)	5.61 (5.16, 6.27)	5.55 (5.16, 6.05)	6.11 (5.50, 7.49)	<0.001
HbA1c (%)	5.50 (5.20, 5.90)	5.50 (5.20, 5.80)	5.90 (5.40, 6.60)	<0.001
MLR	0.27 (0.21, 0.35)	0.26 (0.21, 0.33)	0.30 (0.23, 0.38)	0.002
NLR	1.96 (1.50, 2.65)	1.94 (1.48, 2.59)	2.19 (1.56, 3.09)	0.003
Albumin (g/L)	42.0 (40.0, 44.0)	42.0 (40.0, 44.0)	41.0 (39.0, 44.0)	<0.001
C-reactive protein(mg/dL)	0.20 (0.08, 0.46)	0.18 (0.08, 0.42)	0.25 (0.10, 0.57)	<0.001
CVD mortality, n (%)	903 (6.4%)	377 (3.7%)	526 (16%)	<0.001
All-cause mortality, n (%)	2,519 (18%)	1,121 (11%)	1,398 (43%)	<0.001

Continuous data were presented as median (interquartile range), categorical data were presented as frequencies (percentages). All estimates include a complex survey design.

Abbreviations: CKD, chronic kidney disease; MLR, monocyte-to-lymphocyte ratio; CVD, cardiovascular disease; CHD, coronary heart disease; ACEI, angiotensin-converting enzyme inhibitors; eGFR, estimated glomerular filtration rate; BMI, body mass index; SBP, systolic blood pressure; ALT, Alanine aminotransferase; BUN, blood urea nitrogen; TC, total cholesterol; TG, triglyceride; Non-HDL-C, non-high density lipoprotein cholesterol; HbA1c, glycosylated hemoglobin; NLR, neutrophil to lymphocyte ratio.

### The correlations of MLR and clinical characteristics

The results of partial correlation analysis evaluating the correlations of MLR with clinical characteristics demonstrated that MLR was negatively correlated with eGFR (r = −0.063 (−0.096, −0.034), *p* < 0.001), HbA1c (r = −0.159 (−0.187, −0.135), *p* < 0.001), glucose (r = −0.145 (−0.173, −0.123), *p* < 0.001), TG (r = −0.148 (−0.172, −0.126), *p* < 0.001), TC (r = −0.052 (−0.080, −0.028), *p* = 0.004), red blood cell (r = −0.140 (−0.171, −0.107), *p* < 0.001), hemoglobin (r = −0.087 (−0.114, −0.054), *p* < 0.001), but positively correlated with serum creatine (r = −0.063 0.088 (0.043, 0.140), *p* < 0.001), BUN (*r* = 0.061 (0.024, 0.101), *p* = 0.001) (Table 2).

**Table 2.** The partial correlations of MLR and clinical characteristics.

Variables	MLR	
	r (95% CI)	P-value
Serum creatine	0.088 (0.043, 0.140)	<0.001
eGFR	−0.063 (-0.096, −0.034)	<0.001
BUN	0.061 (0.024, 0.101)	0.001
HbA1c	−0.159 (-0.187, −0.135)	<0.001
Glucose	−0.145 (-0.173, −0.123)	<0.001
TG	−0.148 (-0.172, −0.126)	<0.001
TC	−0.052 (-0.080, −0.028)	0.004
Red blood cell	−0.140 (-0.171, −0.107)	<0.001
Hemoglobin	−0.087 (-0.114, −0.054)	<0.001

The partial correlation analysis adjusted for age, gender, and race.

Abbreviations: MLR, monocyte-to-lymphocyte ratio; eGFR, estimated glomerular filtration rate; BUN, blood urea nitrogen; HbA1c, glycosylated hemoglobin; TC, total cholesterol; TG, triglyceride.

### Associations between MLR and mortality

**Figure 2.**
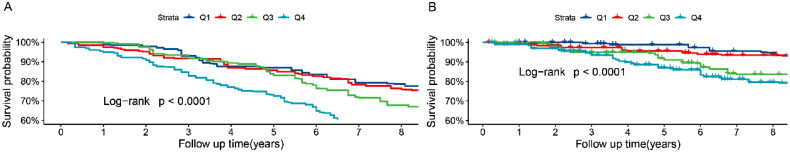
Kaplan-Meier survival curve for mortality by MLR quartiles. Kaplan-Meier survival curve for all-cause mortality (A) and CVD mortality (B) by MLR.

As shown in Table 3, MLR was significantly associated with an increased risk of all-cause mortality (HR = 6.96, 95%CI = 4.24-11.4) in the crude model. After multivariable adjustment, the results remained robust and statistically significant, with model 1 (HR = 4.26, 95%CI = 2.26-8.02), and model 2 (HR = 1.99, 95% CI = 1.10-3.58). Compared to the first quartile of MLR, multivariate-adjusted HRs for patients in the fourth quartile tend to be higher, with crude model (HR = 3.21, 95%CI = 1.93-5.33, P for trend < 0.001), model 1 (HR = 1.96, 95%CI = 1.16-3.31, P for trend = 0.006), model 2 (HR = 1.42, 95%CI = 1.06-3.25, P for trend = 0.014). This statistically significant association was consistent for CVD mortality. The Kaplan–Meier survival curve for all-cause and CVD mortality in the CKD participants stratified by MLR groups was presented in Figure 2. We observed the mortality was higher in individuals with a higher MLR than in those with a lower MLR, which was consistent with cox regression analysis (log-rank *p* < 0.001).

**Table 3.** Weighted association between MLR and mortality.

	MLR					p for trend
	Overall	Q1	Q2	Q3	Q4	
**All-cause mortality**						
Unadjusted	6.96 (4.24, 11.4)	1.00	1.53 (0.79, 2.93)	2.21 (1.26, 3.87)	3.21 (1.93, 5.33)	<0.001
Model 1	4.26 (2.26, 8.02)	1.00	1.32 (0.78, 2.22)	1.90 (1.13, 3.17)	1.96 (1.16, 3.31)	0.006
Model 2	2.72 (1.22, 6.06)	1.00	1.54 (0.84, 2.82)	2.16 (1.26, 3.70)	1.92 (1.10, 3.35)	0.012
**CVD mortality**						
Unadjusted	9.24 (5.86, 14.6)	1.00	1.46 (0.43, 4.91)	2.71 (0.94, 7.81)	4.34 (1.69, 11.2)	0.001
Model 1	5.21 (2.21, 12.3)	1.00	1.15 (0.35, 3.77)	2.21 (0.78, 6.28)	2.45 (0.87, 6.92)	0.044
Model 2	4.19 (1.02, 17.3)	1.00	1.34 (0.32, 5.71)	2.33 (0.70, 7.80)	2.17 (0.71, 6.59)	0.093

Data are presented as HR (95% CI).

Model 1: adjust for age, sex, race, education level, smoking status, hypertension, diabetes, CVD, history of ACEI and stain.

Model 2: adjust for age, sex, race, education level, smoking status, hypertension, diabetes, CVD, history of ACEI and stain, BMI, SBP, TC, HbA1c, BUN, eGFR, CRP, albumin.

Abbreviations: MLR, monocyte-to-lymphocyte ratio; CVD, cardiovascular disease; ACEI, angiotensin-converting enzyme inhibitors; eGFR, estimated glomerular filtration rate; BMI, body mass index; SBP, systolic blood pressure; ALT, Alanine aminotransferase; BUN, blood urea nitrogen; TC, total cholesterol; HbA1c, glycosylated hemoglobin.

### Dose-response relationship between MLR and mortality

**Figure 3.**
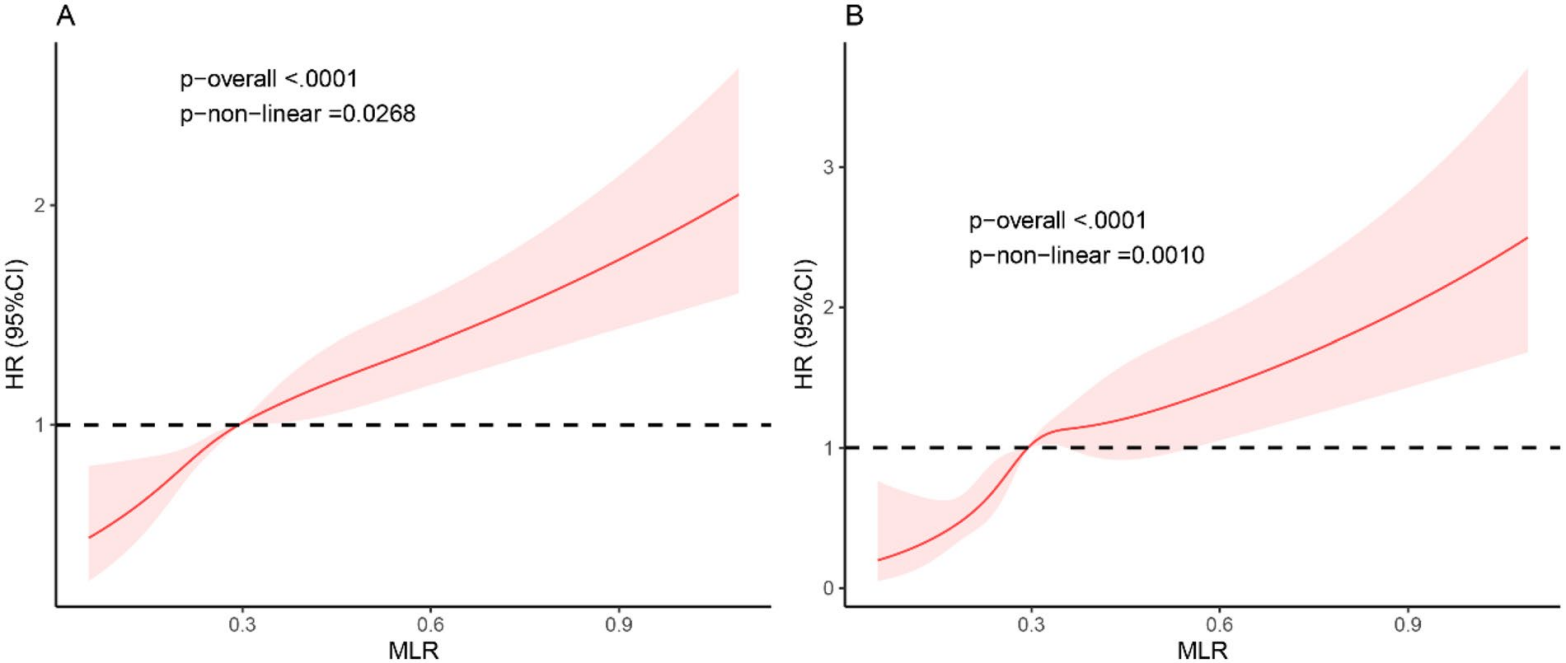
Restricted cubic spline fitting for the association between MLR with mortality. The association of MLR levels with the all-cause (A) and CVD (B) mortality.

Estimated association between MLR and mortality outcomes in CKD population was shown from restricted cubic spline. As demonstrated in Figure 3, after adjusting for multiple potential confounders, we found that the MLR is positively correlated with the risk of all-cause mortality and CVD mortality. The higher the MLR value, the greater the risk of mortality, suggesting that the MLR is an effective clinical indicator for predicting the magnitude of mortality risk.

### ROC analysis of the predictive value of the MLR for all-cause and cardiovascular mortality in CKD

**Figure 4.**
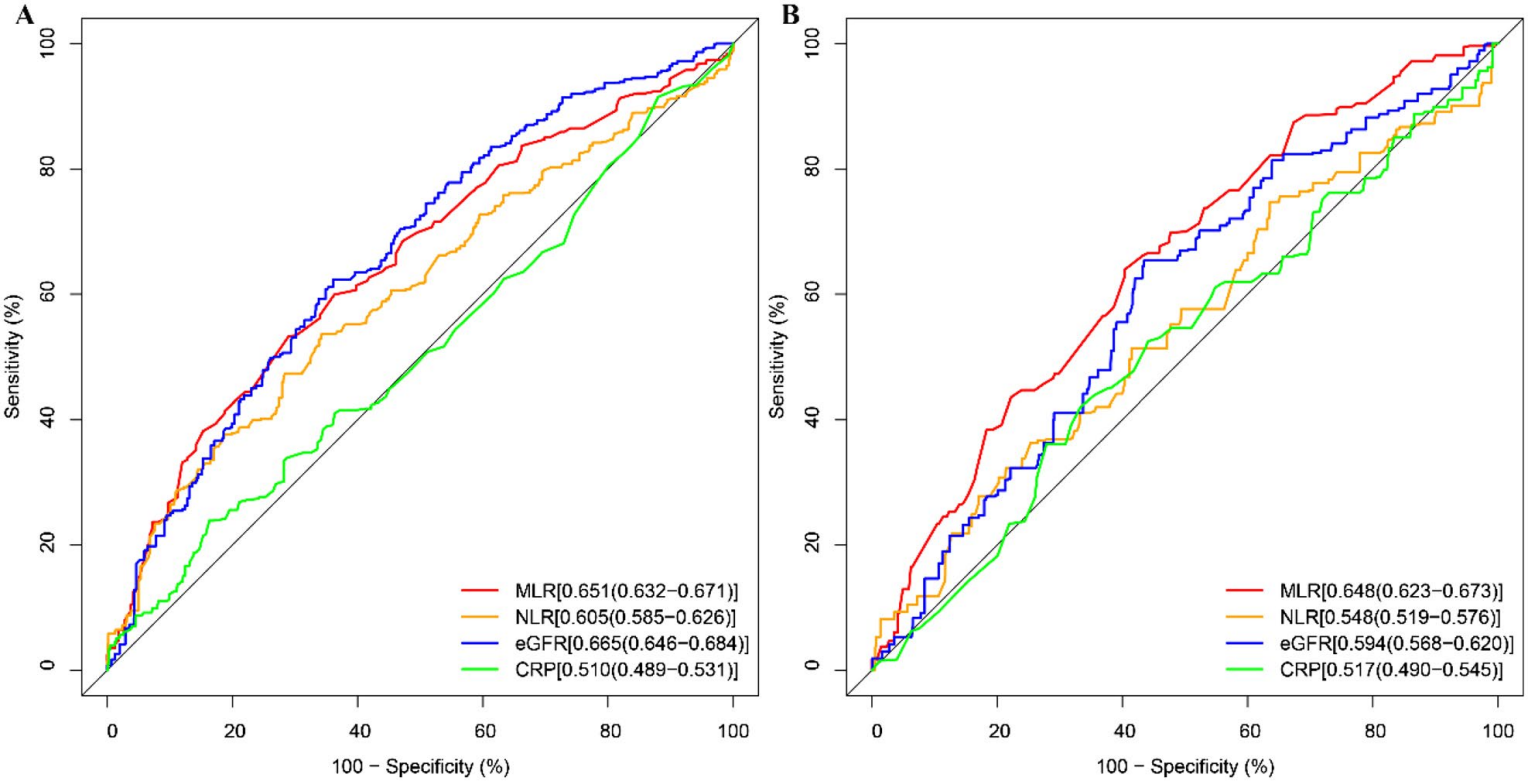
ROC curves of the MLR for predicting mortality. ROC curves and AUC values of the MLR for predicting all-cause mortality (A) and cardiovascular mortality (B)

We compared the efficacy of several clinical indicators in predicting the risk of mortality, including MLR, NLR, CRP, and eGFR (shown in Figure 4). The results indicated that, for the risk of all-cause mortality, the area under the curve (AUC) for MLR (0.651 95%CI: 0.632-0.671) was similar to eGFR (0.665 95%CI: 0.646-0.684), but higher than NLR (0.605 95%CI: 0.585-0.626) and CRP (0.510 95%CI: 0.489-0.531). For the risk of cardiovascular mortality, the predictive ability of MLR (0.648 95%CI: 0.623-0.673) was higher than eGFR (0.594 95%CI: 0.568-0.620), NLR (0.548 95%CI: 0.519-0.576), and CRP (0.517 95%CI: 0.490-0.545) (Table 4).

**Table 4.** The efficacy of MLR in predicting all-cause mortality and CVD mortality.

Variables	AUC	95%CI	cutoff value	Sensitivity	Specificity
**All-cause mortality**					
MLR	65.158	63.194-67.122	0.327	53.29	70.996
NLR	60.566	58.529-62.604	2.416	53.577	65.801
eGFR	66.546	64.626-68.467	58.076	62.303	63.946
CRP	51.004	48.925-53.082	0.715	0.715	0.715
**CVD mortality**					
MLR	64.796	62.286-67.305	0.309	63.878	59.663
NLR	54.791	51.958-57.624	1.811	74.715	36.44
eGFR	59.399	56.792-62.006	57.828	65.399	56.489
CRP	51.762	49.009-54.515	0.165	0.165	0.165

Abbreviation: AUC = areas under the curve; CI = confidence interval, CVD, cardiovascular disease; MLR, monocyte-to-lymphocyte ratio; NLR, neutrophil to lymphocyte ratio; eGFR, estimated glomerular filtration rate; CRP; C-reactive protein.

## Discussion

In this nationwide study, we found that the MLR levels in the CKD population were higher than in the non-CKD population. After adjusting for confounding factors, MLR remained association with the risk of all-cause mortality and cardiovascular mortality. Further analysis revealed a positive correlation between MLR and mortality risk, with MLR demonstrating higher efficacy in predicting mortality risk compared to NLR, eGFR, and CRP. These results confirmed that MLR is a simple and effective clinical indicator for predicting mortality risk in CKD patients.

The occurrence of CKD is usually the result of hypertension, diabetes, and autoimmune diseases, but regardless of the cause, inflammation and activation of the immune system are involved in the development and progression of CKD. Inflammation, through enhancing inflammation or oxidative stress, producing pro-inflammatory cytokines, and altering the immune system, is a significant cause of kidney damage and loss of nephron. Monitoring changes in the levels of inflammation in CKD patients is important. The inflammation marker MLR in routine blood tests is often used to reflect the degree of systemic low-grade inflammation. Previous studies have shown that MLR can predict the onset of CKD [5], and a higher MLR is a strong predictor of mortality risk in end-stage renal disease patients and hemodialysis patients [6–8]. Therefore, we speculate that MLR is an effective biomarker for predicting mortality risk in CKD patients.

Prior studies have been found that monocyte count is a risk factor for cardiovascular mortality and coronary artery plaque formation [9]. Monocytes are important participants in atherosclerosis. Previous investigations supported the correlation between monocyte counts and the severity of atherosclerosis [10] as well as the extent of myocardial infarction [11,12]. The unfavorable remodeling that leads to heart failure is also facilitated by monocytes [13, 14]. Monocyte subtypes (classical, intermediate, and nonclassical) each have a unique role, and intermediate monocytes are associated with reduced ejection fraction and the incidence of cardiovascular events [15, 16]. More and more evidences indicate that intermediate monocytes promote the progression of atherosclerosis in both the general population and CKD patients. These monocytes express a unique chemokine receptor pattern that is associated with atherosclerosis [17]. Intermediate monocytes are prone to secreting pro-inflammatory cytokines, and the increase in monocyte numbers is consistent with deteriorating kidney function [18]. A high monocyte count predicts adverse outcomes in dialysis patients and early-stage CKD patients [19, 20]. It has also been found that the number of intermediate monocyte subpopulations can predict cardiovascular mortality in dialysis patients [21].

Low lymphocyte counts are a frequent occurrence during systemic inflammatory response [22], which may be caused by lymphocyte apoptosis brought on by unchecked immune response [23]. Lower lymphocyte counts are linked to less favorable outcomes in cardiovascular disease patients [24–26]. Lymphocytes mainly consist of B cells, T cells, and natural killer cells. T-cells, further split into T-helper cells (CD4), cytotoxic T-cells (CD8), and regulatory T-cells (Tregs), make up the majority of the lymphocyte population. Each of these cell types has a unique reaction to cardiovascular events. It has been shown that CKD leads to the infiltration of pro-inflammatory T cells in the heart, further damaging myocardial strain, resulting in deterioration of cardiac diastolic function [27]. B lymphocytes have been shown to be associated with ventricular hypertrophy in late-stage CKD patients, potentially contributing to cardiac remodeling in CKD patients [28]. Reduced B1 and B2 cells are significantly negatively correlated with the progression of elderly CKD patients, and the decrease in B cells may be associated with an increased risk of mortality in kidney disease [29].

MLR is calculated based on the number of monocytes and lymphocytes, and can more comprehensively reflect changes in immune status, which are often overlooked by clinical doctors. MLR has already been shown to be a useful indication of the severity and prognosis of cardiovascular disease. A research including 963 non-ST-segment elevation myocardial infarction (NSTEMI) patients found that MLR was a reliable indicator of the degree of coronary lesions and that it was an independent risk factor for major adverse cardiac events (MACE) [30]. MLR has been shown to be independently related with susceptible plaques in individuals with stable angina [31]. High MLR was linked to mortality and MACE in patients with acute coronary syndrome, particularly in younger individuals, according to a meta-analysis. Additionally, it was established that MLR was independently linked to a higher mortality risk in patients with heart failure [32]. Additionally, greater MLR was linked to a higher likelihood of heart failure hospitalization in individuals with coronary artery disease [33]. Previous research has also shown a link between MLR and all-cause mortality in the general population [34], people with a high prevalence of CVD [35], or people with CKD [36].

Previous studies have found that MLR is not only associated with incident CKD, but also related to the risk of mortality in end-stage renal disease and hemodialysis patients. This is consistent with our results, which show that after adjusting for confounders, MLR remains positively correlated with all-cause mortality and cardiovascular mortality risk, and its predictive power is superior to other clinical indicators. This suggests that MLR is a reliable indicator for predicting the risk of death in CKD.

This large-scale study utilized nationwide data and the results are representative. However, our study also has some limitations. Firstly, due to the nature of the investigation, the results are observational rather than causal. Further studies would require prospective research. Secondly, the data used for defining CKD based on creatinine and urine protein levels were single measurements. Lastly, this is a retrospective study, some variables may be subject to recall bias and self-report bias, caution should be exercised when interpreting the results.

## Conclusion

The purpose of this study was to explore the relationship between MLR and the risk of mortality in the CKD population. After adjusting for confounding variables, the level of MLR was positively correlated with the risk of all-cause mortality and cardiovascular mortality. As a convenient and cost-effective inflammatory marker, MLR may play an important role in predicting the risk of death in CKD.

## Ethics approval and consent to participate

The data of this study comes from National Health and Nutrition Examination Survey, Ethics were passed at the beginning of the investigation and the patient provided informed consent.

## Supplementary Material

Supplemental Material

## Data Availability

All analyses during the current study are available in the National Health and Nutrition Examination Survey. This project was supported by the National Natural Science Foundation of China (No. 81870336).
