# Supplementary material for: Association between monocyte-lymphocyte ratio and all-cause and cardiovascular mortality in patients with chronic kidney diseases: A data analysis from national health and nutrition examination survey (NHANES) 2003-2010
Source: Ren Fail. 2024 Jun 4;46(1):2352126. doi: 10.1080/0886022X.2024.2352126 (PMC11151800; doi:10.1080/0886022X.2024.2352126)
Supplement: Supplemental Material [file IRNF_A_2352126_SM4029.docx]

Supplementary table 1 Baseline characteristics of patients with CKD according to quartiles of MLR.

|  | | **MLR** | | | |  |
| --- | --- | --- | --- | --- | --- | --- |
| **Characteristic** | **Overall**, N = 3015 (100%) | **Q1**, N = 633 (21%) | **Q2**, N = 662 (20%) | **Q3**, N = 754 (27%) | **Q4**, N = 966 (32%) | **P Value** |
| Age (years) | 66 (53, 77) | 55 (46, 72) | 62 (47, 74) | 68 (54, 76) | 73 (61, 79) | <0.001 |
| gender |  |  |  |  |  | <0.001 |
| female | 1,492 (54%) | 406 (73%) | 402 (63%) | 379 (54%) | 305 (35%) |  |
| Gender (male, %) | 1,523 (46%) | 227 (27%) | 260 (37%) | 375 (46%) | 661 (65%) |  |
| Race |  |  |  |  |  | 0.029 |
| Mexican American | 496 (6.9%) | 136 (9.8%) | 134 (9.6%) | 108 (5.3%) | 118 (4.6%) |  |
| Non-Hispanic Black | 554 (11%) | 163 (16%) | 165 (15%) | 79 (5.6%) | 147 (8.6%) |  |
| Non-Hispanic White | 1,644 (75%) | 257 (66%) | 298 (67%) | 485 (81%) | 604 (80%) |  |
| Other Race | 321 (7.7%) | 77 (NA%) | 65 (NA%) | 82 (NA%) | 97 (NA%) |  |
| Education level |  |  |  |  |  | 0.4 |
| College or above | 1,058 (42%) | 224 (44%) | 230 (40%) | 260 (47%) | 344 (37%) |  |
| High School | 781 (31%) | 139 (27%) | 142 (28%) | 177 (26%) | 323 (39%) |  |
| Less than high school | 1,176 (27%) | 270 (29%) | 290 (32%) | 317 (27%) | 299 (24%) |  |
| Smoking status (%) |  |  |  |  |  | 0.085 |
| Current smoker | 472 (17%) | 119 (21%) | 128 (19%) | 101 (16%) | 124 (15%) |  |
| Former smoker | 1,028 (30%) | 197 (22%) | 183 (23%) | 227 (27%) | 421 (43%) |  |
| Never smoker | 1,515 (52%) | 317 (57%) | 351 (57%) | 426 (58%) | 421 (41%) |  |
| Comorbidity |  |  |  |  |  |  |
| Hypertension | 2,398 (77%) | 475 (72%) | 511 (70%) | 625 (78%) | 787 (82%) | 0.2 |
| Diabetes | 1,613 (45%) | 399 (49%) | 367 (42%) | 385 (45%) | 462 (44%) | 0.8 |
| CVD | 1,076 (32%) | 166 (19%) | 196 (19%) | 298 (40%) | 416 (42%) | <0.001 |
| Heart failure, n (%) | 380 (11%) | 77 (8.9%) | 53 (5.5%) | 65 (9.7%) | 185 (17%) | 0.13 |
| CHD, n (%) | 422 (14%) | 57 (7.0%) | 69 (7.8%) | 115 (16%) | 181 (21%) | <0.001 |
| Dialysis, n (%) | 61 (9.5%) | 8 (3.9%) | 0 (0%) | 20 (8.4%) | 33 (19%) | 0.4 |
| Medication history |  |  |  |  |  |  |
| Statin | 254 (8.2%) | 52 (7.4%) | 58 (9.2%) | 60 (7.6%) | 84 (8.6%) | 0.5 |
| Aspirin | 68 (2.0%) | 13 (1.3%) | 12 (1.7%) | 17 (2.0%) | 26 (2.8%) | 0.2 |
| ACEI | 205 (6.5%) | 40 (5.9%) | 40 (4.8%) | 57 (7.7%) | 68 (7.1%) | 0.2 |
| ACR (mg/g) |  |  |  |  |  | 0.3 |
| <=30 | 1,008 (35%) | 212 (38%) | 190 (28%) | 217 (31%) | 389 (42%) |  |
| 30-300 | 1,841 (60%) | 396 (59%) | 464 (71%) | 484 (62%) | 497 (52%) |  |
| >=300 | 166 (4.6%) | 25 (3.6%) | 8 (1.4%) | 53 (6.5%) | 80 (5.8%) |  |
| eGFR (ml/min/1.73 m2) |  |  |  |  |  | 0.008 |
| G1 (>90) | 757 (28%) | 261 (43%) | 233 (41%) | 143 (23%) | 120 (14%) |  |
| G2 (60-90) | 605 (19%) | 92 (12%) | 149 (21%) | 166 (20%) | 198 (22%) |  |
| G3a (45-59) | 1,026 (35%) | 154 (28%) | 215 (30%) | 264 (34%) | 393 (44%) |  |
| G3b (30-44) | 372 (11%) | 60 (7.5%) | 57 (6.5%) | 108 (14%) | 147 (12%) |  |
| G4 (15-30) | 211 (6.5%) | 58 (9.8%) | 8 (1.4%) | 63 (7.8%) | 82 (6.5%) |  |
| G5 (<15) | 44 (0.8%) | 8 (0.3%) | 0 (0%) | 10 (0.8%) | 26 (1.7%) |  |
| eGFR (ml/min/1.73 m2) | 59 (51, 92) | 78 (51, 104) | 79 (57, 99) | 57 (47, 85) | 58 (48, 71) | <0.001 |
| BMI (kg/m^2^) | 30 (26, 35) | 32 (27, 37) | 29 (25, 34) | 31 (26, 35) | 29 (24, 33) | 0.3 |
| SBP (mmHg) | 130 (116, 144) | 127 (108, 137) | 128 (116, 144) | 132 (119, 148) | 130 (120, 148) | 0.2 |
| Red blood cell (10^9^/L) | 4.51 (4.07, 4.98) | 4.36 (3.97, 4.99) | 4.64 (4.29, 4.91) | 4.53 (4.17, 4.99) | 4.47 (3.97, 4.98) | 0.2 |
| hemoglobin (g/dL) | 13.90 (12.60, 15.00) | 13.40 (12.40, 14.70) | 14.10 (12.80, 15.10) | 14.10 (12.70, 15.10) | 13.70 (12.50, 14.90) | 0.12 |
| ALT (U/L) | 21 (17, 27) | 18 (15, 26) | 22 (18, 31) | 21 (16, 26) | 21 (16, 27) | 0.027 |
| Serum creatine (umol/L) | 1.06 (0.86, 1.32) | 1.02 (0.80, 1.20) | 0.99 (0.79, 1.22) | 1.10 (0.90, 1.32) | 1.12 (0.95, 1.40) | <0.001 |
| BUN (mmol/L) | 6.07 (4.28, 8.21) | 5.36 (3.57, 6.78) | 5.71 (4.28, 7.50) | 6.07 (4.64, 8.57) | 7.14 (4.64, 8.93) | 0.018 |
| TC (mg/dL) | 180 (153, 206) | 189 (145, 211) | 184 (159, 209) | 186 (156, 211) | 168 (143, 188) | 0.002 |
| TG (mg/dL) | 127 (92, 197) | 143 (108, 234) | 140 (103, 205) | 142 (97, 209) | 114 (82, 156) | 0.002 |
| Non-HDL-C (mg/dL) | 126 (100, 157) | 134 (92, 163) | 126 (107, 164) | 136 (111, 163) | 112 (91, 141) | 0.001 |
| Glucoses (mmol/l) | 6.11 (5.50, 7.49) | 6.11 (5.50, 7.94) | 6.22 (5.61, 8.16) | 6.11 (5.33, 8.16) | 6.05 (5.50, 6.77) | 0.3 |
| HbA1c (%) | 5.90 (5.40, 6.60) | 5.80 (5.30, 6.50) | 5.80 (5.50, 6.80) | 5.90 (5.50, 6.71) | 5.90 (5.40, 6.50) | >0.9 |
| NLR | 2.19 (1.56, 3.09) | 1.55 (1.13, 2.01) | 1.92 (1.46, 2.48) | 2.07 (1.59, 2.77) | 3.23 (2.37, 4.00) | <0.001 |
| Albumin (g/L) | 41.0 (39.0, 44.0) | 40.0 (38.0, 43.0) | 42.0 (39.0, 43.0) | 41.0 (39.0, 44.0) | 42.0 (39.0, 44.0) | 0.4 |
| C-reactive protein (mg/dL) | 0.25 (0.10, 0.57) | 0.26 (0.14, 0.57) | 0.26 (0.12, 0.53) | 0.26 (0.10, 0.49) | 0.21 (0.10, 0.52) | 0.6 |
| CVD mortality, n (%) | 526 (16%) | 53 (7.1%) | 85 (10%) | 143 (18%) | 245 (25%) | 0.009 |
| All-cause mortality, n (%) | 1,398 (43%) | 197 (23%) | 244 (34%) | 341 (47%) | 616 (59%) | <0.001 |
| Continuous data were presented as median (interquartile range), categorical data were presented as frequencies (percentages). All estimates include a complex survey design. | | | | | | |
| Abbreviations: CKD, chronic kidney disease; MLR, monocyte-to-lymphocyte ratio; CVD, cardiovascular disease; CHD, coronary heart disease; ACEI, angiotensin-converting enzyme inhibitors; eGFR, estimated glomerular filtration rate; BMI, Body mass index; SBP, systolic blood pressure; ALT, Alanine aminotransferase; BUN, blood urea nitrogen; TC, total cholesterol; TG, triglyceride; Non-HDL-C, non-high density lipoprotein cholesterol; HbA1c, glycosylated hemoglobin; NLR, neutrophil to lymphocyte ratio. | | | | | | |
